# Biatrial thrombi resembling myxoma regressed after prolonged anticoagulation in a patient with mitral stenosis: a case report

**DOI:** 10.1186/s13256-016-1018-0

**Published:** 2016-08-10

**Authors:** Hou Tee Lu, Rusli Nordin, Norliza Othman, Chun Ngok Choy, Ji Yen Kam, Benjamin Cheang-Leng Leo, Gunasekaran Ramsamy, Teck Hwa Goh

**Affiliations:** 1 Clinical School Johor Bahru, Jeffrey Cheah School of Medicine and Health Sciences, Monash University Malaysia, 8 Jalan Masjid Abu Bakar, 80100 Johor Bahru, Johor Malaysia; 2 Department of Radiology, Sultanah Aminah Hospital, Jalan Abu Bakar, 80100 Johor Bahru, Johor Malaysia; 3 Department of Cardiology, Sultanah Aminah Hospital, Jalan Abu Bakar, 80100 Johor Bahru, Johor Malaysia; 4 Department of Cardiology, Penang General Hospital, Jalan Residensi, 10990 Georgetown, Pulau Pinang Malaysia

**Keywords:** Cardiac mass, Thrombus, Myxoma, Atrial fibrillation, Mitral stenosis, Echocardiography, Cardiac magnetic resonance

## Abstract

**Background:**

Many cases of cardiac masses have been reported in the literature, but in this case report we described a rare case of biatrial cardiac mass that represented a challenge for diagnosis and therapy. The differentiation between cardiac masses such as thrombi, vegetations, myxomas and other tumors is not always straightforward and an exact diagnosis is important because of its distinct treatment strategy. Transthoracic/esophageal echocardiography and cardiac magnetic resonance play an important role in establishing the diagnosis of cardiac masses. However, no current noninvasive diagnostic tool has the ability to absolutely diagnose cardiac masses; obtaining a pathological specimen by surgical resection of cardiac masses is the only reliable method to diagnose cardiac masses accurately. Our case report is an exception in that the final diagnosis was affirmed by empirical anticoagulation therapy based on clinical judgment and noninvasive characterization of biatrial mass.

**Case presentation:**

We described a 54-year-old Malay man with severe mitral stenosis and atrial fibrillation who presented with a biatrial mass. Transthoracic/esophageal echocardiography and cardiac magnetic resonance detected a large, homogeneous right atrial mass typical of a thrombus, and a left atrial mass adhering to interatrial septum that mimicked atrial myxoma. The risk factors, morphology, location, and characteristics of the biatrial cardiac mass indicated a diagnosis of thrombi. However, our patient declined surgery. As a result, the nature of his cardiac masses was not specified by histology. Of note, his left atrial mass was completely regressed by long-term warfarin, leaving a residual right atrial mass. Thus, we affirmed the most probable diagnosis of cardiac thrombi. During the course of treatment, he had an episode of non-fatal ischemic stroke most probably because of a thromboembolism.

**Conclusions:**

Noninvasive characterization of cardiac mass is essential in clarifying the diagnosis and directing treatment strategy. Anticoagulation is a feasible treatment when the clinical assessment, risk factors, and imaging findings indicate a diagnosis of thrombi. After prolonged anticoagulation therapy, complete resolution of biatrial thrombi was achievable in our case.

## Background

The differentiation between cardiac masses such as thrombi, vegetation, myxomas, and other tumors is not always straightforward and an exact diagnosis is important because of its distinct treatment strategy [1]. Transthoracic echocardiography (TTE) and transesophageal echocardiography (TEE) play an important role in establishing a diagnosis of cardiac masses [2]. Cardiac magnetic resonance (CMR) offers potential advantages and is complementary to echocardiography in the evaluation of cardiac masses [3, 4]. However, no current noninvasive diagnostic tool has the ability to absolutely diagnose cardiac masses; obtaining the pathological specimen by surgical resection of cardiac masses is the only reliable method to diagnose cardiac masses accurately [5–7].

## Case presentation

In January 2011, a 54-year-old Malay man was referred to our hospital for evaluation of palpitation. On examination, his pulse rate was 85/minute with irregular rhythm and his blood pressure was 120/80 mmHg. He was afebrile. A loud first heart sound and a soft mid-diastolic rumbling murmur (grade 2/6 degrees) were auscultated at mitral area with no signs of heart failure. Electrocardiography revealed atrial fibrillation (AF). The laboratory results showed normal hemoglobin, white cells, and platelet counts. Renal test, liver function test, and other laboratory test results were unremarkable. His urine analysis was also normal.

**Fig. 1:**
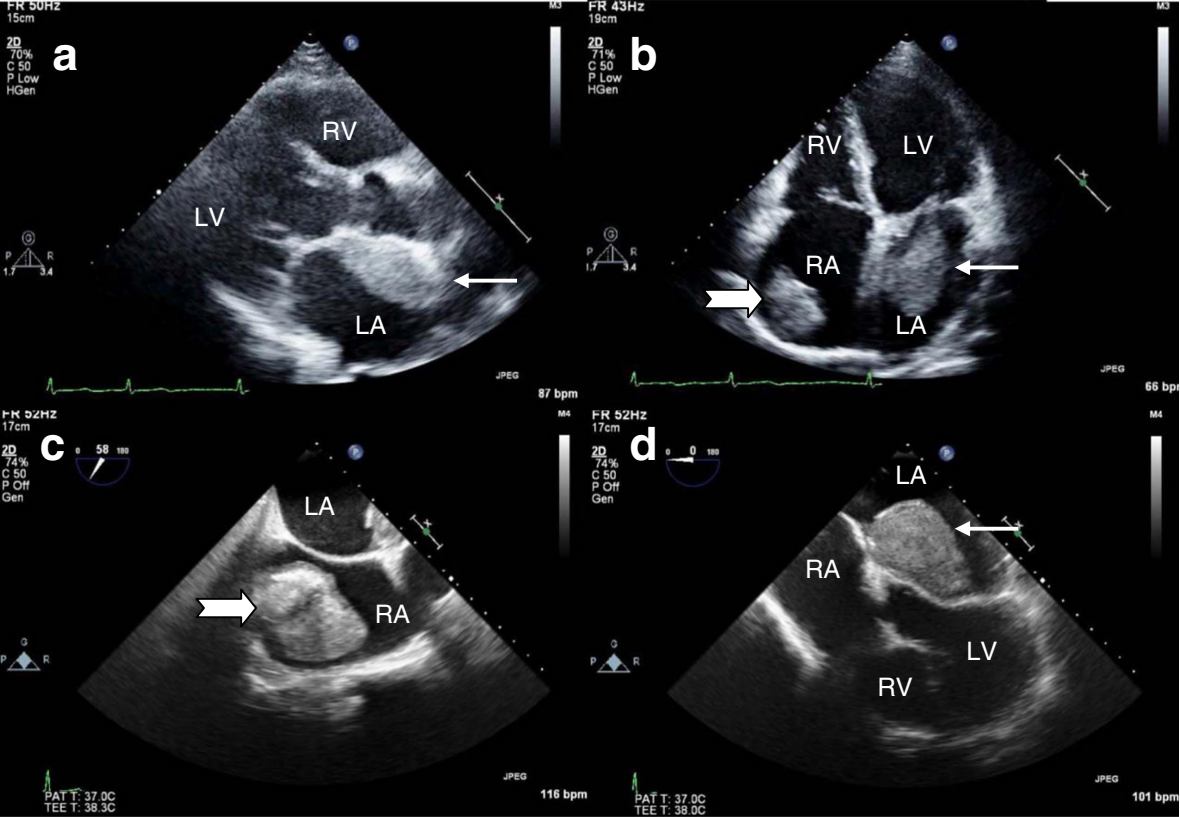
Images obtained by transthoracic echocardiography and transesophageal echocardiography. **a**, **b** Transthoracic echocardiography images. **c**, **d** Transesophageal echocardiography images. A large left atrial mass (*arrows*) adhering to interatrial septum in parasternal long axis (**a**) and apical four chamber view (**b**, **d**) and a giant right atrial mass (*notched arrows*) was seen in apical four chamber (**b**) and bicaval view (**c**). *LA* left atrium, *LV* left ventricle, *RA* right atrium, *RV* right ventricle

TTE and TEE revealed severe mitral stenosis (MS). His mitral valve was thickened and moderately calcified, and his anterior mitral valve leaflet was “dooming” during diastole suggestive of rheumatic origin. His mitral valve area, which was estimated by two-dimensional planimetry and pressure half-time method, was 0.92 cm^2^ and 0.91 cm^2^, respectively. His peak and mean mitral valve gradients were 14 and 9 mmHg, respectively. His left atrium (LA) and right atrium (RA) were dilated. In his RA, we found a large, mobile, homogeneous round mass measuring 40×35 mm. A large LA mass with similar echogenicity to his RA mass was found adhering to interatrial septum and protruding into the left ventricle with cardiac motion (Fig. 1). We could not identify any stalk attached to the LA mass by TTE and TEE examinations. In addition, there were no identifiable masses in the left atrial appendage. Spontaneous echocardiographic contrast (SEC) was observed in his LA and left ventricle. Mild tricuspid regurgitation and mild pulmonary hypertension were identified with a peak pulmonary systolic pressure of 26 mmHg (estimation based on a peak tricuspid regurgitation jet velocity of 210 cm/second and an estimated right atrial pressure of 5 mmHg). His inferior vena cava was not dilated and collapsible with respiration, and there was no thrombus. His ejection fraction was estimated as 45 %.

**Fig. 2:**
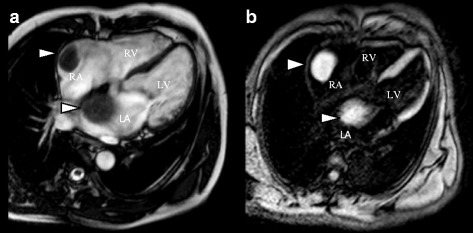
Cardiac magnetic resonance. Cine four chamber view: “white blood” gradient echo (**a**) and “black blood” imaging (**b**) on early gadolinium images showed a low signal floating lesion in the right atrium and a left atrial lesion adhering to the interatrial septum (*white triangles*). *LA* left atrium, *LV* left ventricle, *RA* right atrium, *RV* right ventricle

Patients with severe MS and AF are at high risk of developing intracardiac thrombi. Based on the findings by TTE, TEE, and the presence of risk factors (AF), his RA mass was likely to be a thrombus as described earlier. Similar features were found in LA mass and it was thought to be a thrombus as well. However, the characteristic of LA mass adhering to atrial septum mimics atrial myxoma, which is the most common benign cardiac tumor [1]. The shape, mobility, and location of the LA mass made it difficult to rule out atrial myxoma with absolute certainty. Therefore, our patient underwent CMR for additional noninvasive characterization of the biatrial mass. CMR showed a giant right atrial mass measuring 5.3×3.2×3.9 cm and a large left atrial lesion measuring 5.0×2.4×5.1 cm adhering to interatrial septum (Fig. 2). Both lesions were intermediate in signal on cine CMR, and not enhanced during early and delayed enhancement CMR. The morphology, location, and avascular characteristics made thrombi the most likely diagnosis. The CMR result was in agreement with TTE and TEE findings.

**Fig. 3:**
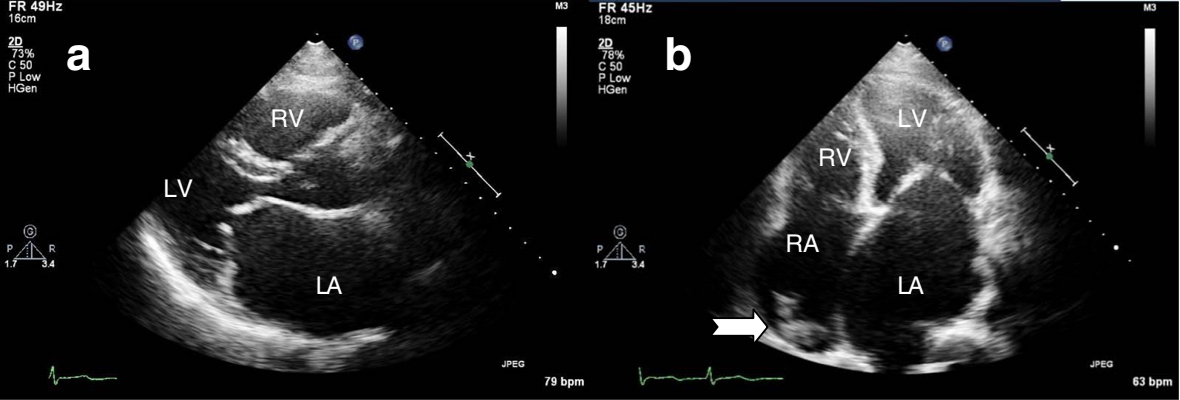
Outcome after long-term anticoagulation therapy. The left atrial thrombus is completely resolved as viewed from parasternal long axis (**a**) and apical four chamber view (**b**) in the left atrium. A residual right atrial thrombus (*notched arrow*) is still visible in the right atrium (**b**). *LA* left atrium, *LV* left ventricle, *RA* right atrium, *RV* right ventricle

Based on his history, physical examinations and imaging findings, we recommended thrombectomy and mitral valve replacement. Despite medical advice, our patient declined surgery. We empirically initiated heparin and warfarin administered intravenously to prevent thromboembolism. His international normalized ratio (INR) was targeted at 2.5 to 3.5. Bisoprolol was prescribed for AF rate control. However, his clinical course was complicated by his non-adherence to warfarin. His INR was subtherapeutic (median=1.8). After 2 years of warfarin (partial compliance), serial TTE showed no regression of LA and RA masses. After 3 years of uneventful follow-up, he experienced an episode of transient ischemic attack in February 2014. Subsequently, in December 2014, he had an episode of non-fatal ischemic stroke manifested by right hemiparesis (motor power reduced to 3/5) most probably because of thromboembolism. We counseled him with regard to the danger of the thromboembolism and re-emphasized the need for treatment compliance. Fortunately, he resumed warfarin and did not demonstrate further thromboembolic events. In March 2016 (5 years after the detection of biatrial masses), a follow-up TTE documented complete regression of the LA mass and a near-complete resolution of the RA mass (Fig. 3). Thus, the most probable diagnosis of cardiac thrombi was affirmed. He remains well on medical treatment.

## Discussion

Many cases of cardiac masses have been reported in the literature, but in this case report we described a rare case of biatrial cardiac mass that represented a challenge for diagnosis and therapy. In our patient, the characteristic of a left atrial mass adhering to the atrial septum posed a diagnostic challenge in differentiating between thrombus and myxoma, the most commonly reported cardiac masses. The following discussion will review cases of biatrial masses in patients with MS reported between 2008 and 2016 (Table 1). First, a 40-year-old woman with rheumatic MS was found to have biatrial thrombi mimicking myxoma, and she underwent a successful thrombectomy and valve replacement [8]. Second, a 77-year-old man with AF, severe MS and heart failure presented with dyspnea. TEE and TTE revealed biatrial thrombi confirmed by pathological examination following thrombectomy and mitral valve replacement [9]. Third, a 58-year-old woman presented with acute limb ischemia; she was found to have mobile biatrial thrombi, AF, and MS and underwent successful embolectomy, thrombectomy, and mitral valve replacement [10]. The features of biatrial thrombi, MS, and AF were common to all three patients. In addition, a case of biatrial myxoma with mild MS presented with cerebral ischemia was successfully treated with thrombolytic therapy administered intravenously and surgical resection [11]. However, numerous cases of cardiac mass with or without mitral valve disease with diagnostic difficulties have been reported earlier. Overall, cardiac thrombi were frequently reported [5, 7, 10, 12, 13]. In some instances a cardiac thrombi mimicked atrial myxoma [5, 7]. By contrast, atrial myxoma can simulate thrombus in the setting of MS [14]. Of interest, a thrombus could form on top of a myxoma. In one case report of a patient with MS, the left atrial mass showed features of thrombus characterized by echocardiography and CMR. However, histopathological evaluation of the left atrial mass removed during surgery revealed a massive thrombus formed on top of a very small pre-existing left atrial myxoma [12]. To complicate matters further, atrial thrombus may have a stalk [6] or neovascularization [13] mimicking atrial myxoma, potentially leading to a delay in anticoagulation therapy. In another case report, biatrial intracardiac masses were detected by three-dimensional TEE in an 80-year-old woman with heart failure, mitral valve repair, dual chamber permanent pacemaker implantation, and AF. Although direct pathological specimens were not obtained, the reduction in the size of both masses after intensive anticoagulation treatment raises the strong possibility that both masses were thrombi [15]. This case report highlighted the facts that anticoagulation is a feasible treatment in regressing thrombi when surgery is relatively contraindicated in an older patient with comorbidities.

**Table 1:** Biatrial mass with mitral stenosis

Author	Year	Age/Sex	Presentation	Atrial fibrillation	Diagnosis	Treatment
Ibrahim *et al*. [11]	2008	51/male	Right hemiparesis	–	Myxoma	Alteplase administered intravenously + tumor excision
Tasdemir *et al*. [10]	2008	58/female	Left foot pain	+	Thrombi	Thrombectomy + MV surgery
Tsubokawa *et al*. [9]	2010	77/male	Dyspnea	+	Thrombi	Thrombectomy + MV surgery
Khanna *et al*. [8]	2015	40/female	Dyspnea	+	Thrombi	Thrombectomy + MV surgery

*MV* mitral valve, (+) presence, (–) absence

TEE is superior to TTE in delineating and characterizing cardiac masses [2]. CMR provides high spatial resolution images, improves tissue characterization and is complementary to echocardiography in the assessment of cardiac masses [3]. Common CMR sequences are cine image, T1-weighted and T2-weighted spin echo, contrast first pass perfusion, and standard delay enhancement. However, tumors and chronic organized thrombi cannot be distinguished from one another using the morphology, motility, and enhancement patterns by CMR. A pattern of hyperintensity/isointensity (compared with normal myocardium) with short T1, and hypointensity with long T1, was very frequent in thrombi, rare in tumors, and had the highest accuracy for the differentiation of both entities [4]. In addition to conventional imaging studies, the assessment of vascularity either by myocardial perfusion contrast echocardiography [16] or cardiac catheterization may assist in the differentiation of thrombi and other type of cardiac tumors. Nevertheless, in a few instances, the final diagnosis of cardiac masses can only be made by obtaining a pathological specimen after surgical resection of cardiac masses [5–7, 12].

For our patient, the nature of biatrial mass was not specified by histology because he declined surgery. The presence of MS, AF, SEC and dilated LA indicated a diagnosis of thrombi. Our case is unusual because the final diagnosis was affirmed by empirical anticoagulation based on clinical judgment and noninvasive characterization of biatrial mass. A reported case of left atrial thrombus with a stalk showed that a trial of anticoagulation was beneficial in regressing the cardiac mass particularly when the differential diagnosis was difficult and thrombus was a possibility [6]. There were also cases that reported successful regressions of the thrombus with anticoagulation without the need for surgical thrombectomy [15, 17, 18]. Regression of thrombus using warfarin has been studied in patients with MS and left atrial thrombus detected prior to percutaneous transvenous mitral commissurotomy. Among 219 patients following 6 months of warfarin (INR 2 to 3) therapy, 24.2 % of patients were found to have complete resolution of thrombus and 75.8 % of patients were found to have partial resolution of thrombus, and a higher INR (at least 2.5) predicted thrombus resolution [19]. For our patient, the regression of his cardiac masses was not observed in the short term after treatment with warfarin, casting doubt on the true identity of the biatrial mass. We suspect the main contributing factor towards the lack of thrombi regression was inadequate anticoagulation owing to his lack of compliance. For this reason, compliance to anticoagulation therapy was pivotal in thrombi regression, and it took 5 years to visualize the resolution of biatrial thrombi. Although a thorough attempt had been made, we could not convince our patient to agree to surgery. We believe that early mitral valve surgery and thrombectomy is beneficial in preventing thromboembolism.

## Conclusions

Noninvasive characterization of cardiac mass is essential in clarifying the diagnosis and directing treatment strategy. Anticoagulation is a feasible treatment when the clinical assessment, risk factors, and imaging findings indicate a diagnosis of thrombi. After prolonged anticoagulation therapy, complete resolution of biatrial thrombi was achievable in our case.

## Abbreviations

AF, atrial fibrillation; CMR, cardiac magnetic resonance; INR, international normalized ratio; LA, left atrium; MS, mitral stenosis; RA, right atrium; SEC, spontaneous echocardiographic contrast; TEE, transesophageal echocardiography; TTE, transthoracic echocardiography
